# Case report: Successful anterior temporal lobectomy in drug-resistant temporal lobe epilepsy associated with Sotos syndrome

**DOI:** 10.3389/fneur.2023.1126327

**Published:** 2023-03-09

**Authors:** Leonardo Favi Bocca, Thiago Pereira Rodrigues, Thiago Bortholin, Elza Márcia Targas Yacubian, Henrique Carrete Júnior, Mirian Guaranha, Ricardo Silva Centeno

**Affiliations:** ^1^Department of Neurology and Neurosurgery, Federal University of São Paulo, São Paulo, Brazil; ^2^Department of Diagnostic Imaging, Federal University of São Paulo, São Paulo, Brazil

**Keywords:** Sotos syndrome, temporal lobe epilepsy, epilepsy surgery, outcome, hippocampal sclerosis

## Abstract

The Sotos syndrome is an autosomal dominant disorder characterized by haploinsufficiency of *NSD1* gene, with some individuals affected by epilepsy and, rarely, drug-resistant seizures. A 47-years-old female patient with Sotos syndrome was diagnosed with focal-onset seizures in left temporal lobe, left-side hippocampal atrophy, and neuropsychological testing with decreased performance in several cognitive domains. Patient was treated with left-side temporal lobe resection and developed complete awake seizure control in 3-years of follow-up, with marked improvement in quality-of-life. In selected, clinically concordant patients, resective surgeries may play a significant role in improving patient's quality of life and seizure control.

## Introduction

The Sotos syndrome (OMIM #117550), previously named as cerebral gigantism, is an overgrowth syndrome (1), first described in 1964 by Sotos et al. (2). This condition is characterized by childhood overgrowth and comprises three cardinal features: childhood overgrowth (with significant macrocephaly), characteristic facial appearance (high, broad forehead, fronto-temporal hair sparsity, malar flushing, down-slanting palpebral fissures, and a pointed chin) and learning difficulties (3). Other major features may be present and include advanced bone age, poor feeding in infancy, neonatal jaundice, neonatal hypotonia, seizures, scoliosis, cardiac anomalies, renal anomalies, maternal pre-eclampsia, and joint laxity/pes planus (3).

The pathogenic haploinsufficiency of the *N*uclear receptor *S*et *D*omain containing protein 1 gene (*NSD1*) was found as major cause of Sotos syndrome (4). Mutations in *NSD1* gene or 5q35 microdeletions encompassing *NSD1* are the most common etiology (5). The condition is inherited in an autosomal dominant manner, with ~95% of individuals presenting with *de novo* pathogenic variant (6).

The presentation of seizures is common with around half of Sotos patients having at least one episode (7), with wide range of possible presentations reported in the literature. A recent cohort published by Dassi et al. (8) described the phenotype of seizures in 49 patients, with 10% showing only febrile seizures, and 90% with clear epilepsy. Among all seizure types, staring spells (both true generalized onset absence seizures and focal onset impaired awareness seizures) was the most frequent one, encompassing 67% of reported patients.

Although surgical treatment of drug-resistant seizures in one patient with Sotos syndrome was reported (9), to the best of authors' knowledge, this is the first successful surgical treatment of a genetically confirmed Sotos syndrome affected patient in the literature.

## Case presentation

### Patient information

A 47-years-old white female patient was referred to our institutional epilepsy clinic with a history of seizures since 2 years-old. The first epileptic event was described as sudden loss of awareness, followed by generalized clonic movements and sialorrhea with no fever or other precipitating symptom. The first antiseizure drug trial was phenobarbital. Despite correct dose and usage of antiseizure medications, the patient continued to experience seizures since 2-years old. From 20-years-old to our first clinical evaluation, the patient experienced a focal onset impaired awareness seizure, characterized by an aura of desire to cough or a feeling of dry throat and followed by staring, mouth automatisms, bimanual automatisms, and head shift to the left side, evolving to postictal state lasting about 2 min. Overall seizure time was brief, lasting around 1 min and secondary generalizations rarely happening, with only four events during lifetime. This awake seizure had a minimal frequency of two to three seizures by week, worsening frequency close to menses phase, with no history of status epilepticus. Patient's mother reported a second type of event described as agitation and frenetic rubbing of left ear with ipsilateral hand at sleeping time, with an average frequency of one to two events each week. Previous history of several antiseizure drug trials included valproate, phenytoin, lamotrigine and phenobarbital, with variable degrees of tolerance and seizure control. Current antiseizure medication was carbamazepine and clobazam, without seizure free achievement. Remarkable past medical history included no perinatal disorder, non-consanguineous parents and no history of epilepsy or seizures in the family.

### Clinical findings

**Figure 1:**
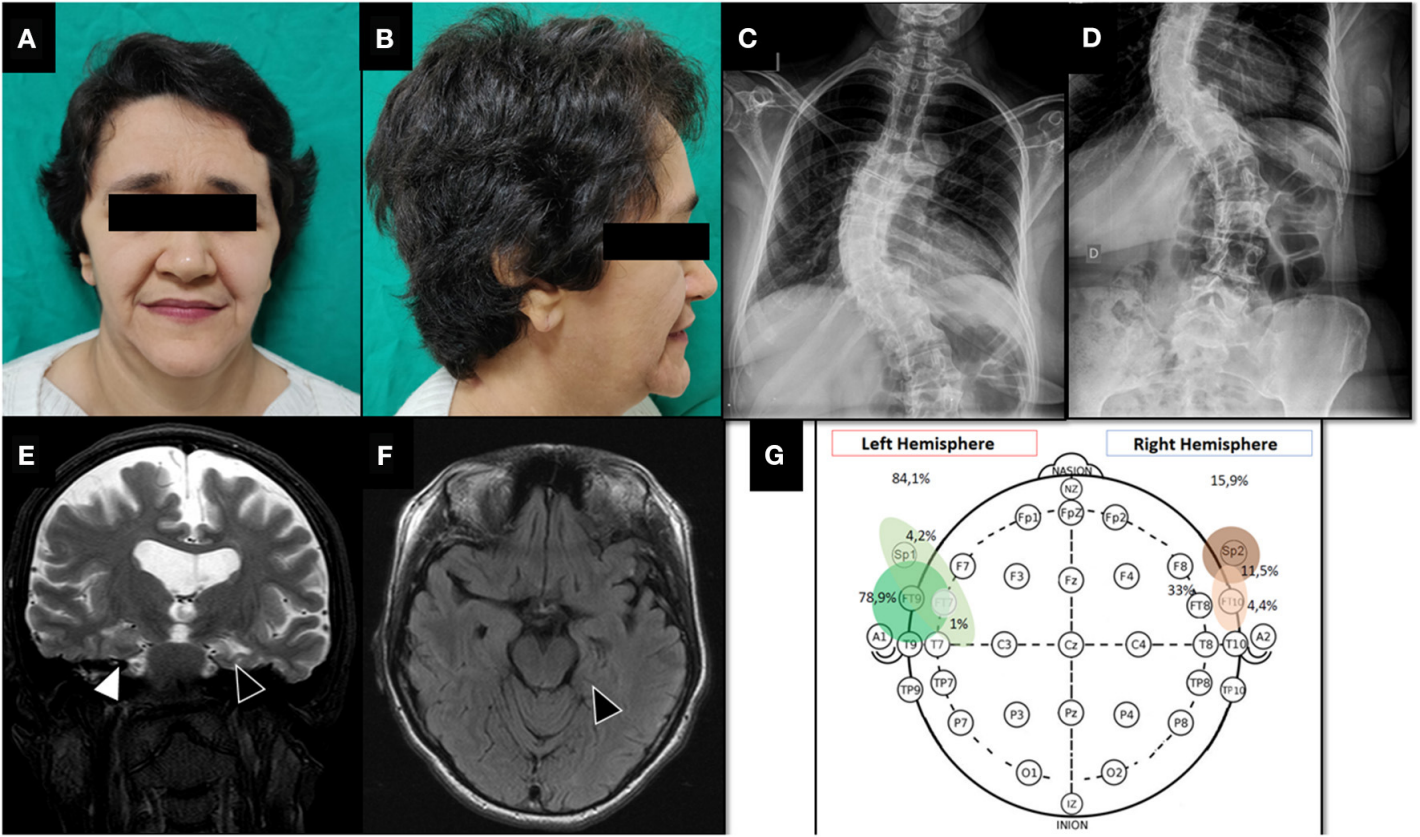
Pre-surgical evaluation. **(A, B)** Facial dysmorphic features of Sotos syndrome showing prominent forehead, pointed chin and macrodolicocephaly. **(C, D)** Plain spine radiograph shows thoraco-lumbar scoliosis. **(E)** T2-weighted coronal brain MRI showing global atrophy with marked sulcal and ventricular volume increase. Note left hippocampal malrotation/atrophy (black triangle) compared to right hippocampus (white triangle). **(F)** FLAIR-weighted axial brain MRI. Left hippocampal malrotation/atrophy with slight hyperintense signal (black triangle). **(G)** Interictal EEG monitoring showing bilateral epileptic discharges with left temporal lobe lateralization.

Patient's family reported learning disabilities and phenotypic features of Sotos syndrome (Figure 1) in clinical and imaging exams were present. The genetic testing confirmed the diagnosis showing a heterozygous mutation in exon 15 of *NSD1* gene [c.5146G>A, p.(Gly1716Arg)]. This variant was not found in The Genome Aggregation Database (gnomAD) and was classified as pathologic in Leiden Open Variation Database (LOVD) (10).

Prolonged (62 h) non-invasive video-EEG monitoring was performed on 32-channel digital EEG equipment (Ceegraph software, Bio-Logic Systems Corp., Mundelein, IL, U.S.A. and QP-110AK Nihon Kohden, Tokyo, Japan). Electrodes were placed according to the 10–20 International System, plus intermediary temporal and sphenoidal electrodes. To record ictal events, antiseizure medications were completely withdrawn. The frequency and location of interictal epileptiform discharges (IEDs) were visually assessed on 5 min EEG samples per hour, 24 h per day. This monitoring showed symmetrical and slightly disorganized base activity, with 778 bilateral interictal epileptic discharges (84.1% left anterior temporal region and 15.9% right anterior temporal region, Figure 1G) and six electroclinical seizures. Five focal onset impaired awareness motor seizures were during awake period and only one during sleep.

All seizures recorded had the ictal onset and sytomatogenic area in the left temporal lobe, and brain MRI showed left hippocampus atrophy and right hippocampus malrotation (Figure 1). Neuropsychological testing found low scores in episodic memory (verbal and non-verbal), language, speech, constructive praxis, and working memory. Quality of life was assessed using Quality of Life in Epilepsy Inventory-31 (QOLIE-31) adapted to patient's native language (11), with a pre-operative score of 45.4.

### Therapeutic intervention

Left anterior temporal lobectomy was performed (En bloc resection of the 4.5 cm anterior region of temporal neocortex, piecemeal resection of uncus, amygdala and entorhinal cortex and en bloc resection of the anterior 2.5 cm of the hippocampus) by the senior epilepsy neurosurgeon (RSC). The patient was discharged from the hospital after 3 days, with no complication.

### Follow-up and outcomes

**Figure 2:**
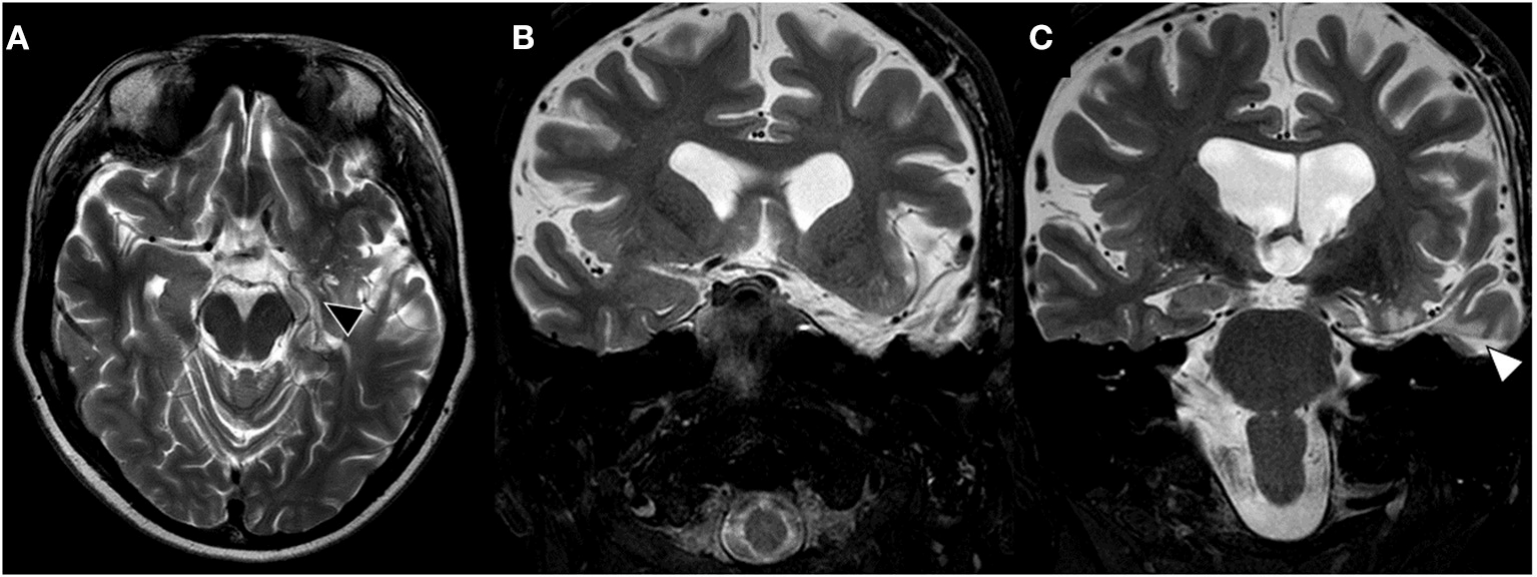
Late post-surgical brain MRI. **(A)** T2-weighted axial brain MRI shows left hippocampus resection (black triangle). **(B, C)** Coronal brain images showing complete resection of left amygdala and uncus on image **(B)** and hippocampal resection through transcortical approach (T2, white triangle).

During 3-years follow-up, the patient is under previous antiseizure medication dosage (carbamazepine 400 mg q8h and clobazam 20 mg q12h), with post-operative brain MRI showing complete hippocampal and temporal resection (Figure 2). Patient had no epileptic event awake, experiencing on average one nocturnal seizure each week (Engel Outcome Scale class II D).

Two awake EEG performed at 2- and 21-month after surgery showed no awake interictal epileptic discharges. Post-operative QOLIE-31 score was 63.3 (an improvement of 39.4%), and neuropsychological assessment showed no change in constructive praxis, language, speech, and verbal episodic language, and slight improvement of non-verbal episodic memory. Despite no change in verbal episodic memory, patient described subjective worsening in this cognitive domain.

## Discussion

Heterogenous seizure patterns have been associated with Sotos syndrome, e.g., febrile seizures, infantile spasms, absence, tonic–clonic, and myoclonic seizures (12), occurring in 15%−50% of affected patients (3, 7) and a wide spectrum of behavioral and emotional disturbances (e.g., attention-deficit-hyperactivity disorder, aggressiveness, irritability, pyromania, social inhibition, psychosis, and autistic features) are commonly related to patients with this condition (5). Seizure-free status after one or more drug trials is the rule (12), with persistence of seizures in adulthood, i.e., drug-resistant epilepsy, an uncommon (9%) outcome (13). Around 40% of Sotos syndrome patients have a classic temporal lobe seizure (abdominal auras, automatisms with/without behavioral arrest and a temporal onset on ictal EEG), with tonic-clonic generalization developing in one third of patients (12). EEG data from big cohorts shows different characteristics, ranging from generalized, focal, or multifocal epilepsy (8). The heterogeneity in clinical behavior, genetic mutation (8) and seizure control must guide detailed evaluation by the attending clinician.

Although the goal of resective temporal lobe surgery for epilepsy treatment is to achieve seizure-free status, and with some big, published cohorts showing rates of chronic control (Engel Outcome Scale class I) ranging from 62 to 73.6% (14–16), the quality-of-life improvement impact is observed even in patients with Engel outcome class >I (17), probably due to the multifactorial effect of seizure control in different cognitive aspects (like mood, medication use/dosage, social function, etc.).

Predictors of better outcome after epilepsy surgery include congruent electrophysiology data, lesional epilepsy, and surgical limitations to first epilepsy surgery (18). Even after recurrence of seizures, second look surgery can still be a possible therapeutic option, with reported rates of 57% of seizure free status after first surgery failure (19). The patient presented in this report continued to experience sleep seizures despite complete mesial temporal lobe resection, what may be due to partial epileptogenic zone resection. Although still presenting with nocturnal seizures, the patient and his family reported significant improvement in quality of life, enabling patient's return to unattended work.

Genetic testing is not routinely performed before surgical evaluation in most epilepsy centers and is not included in suggested presurgical list of tests of Epilepsy League Against Epilepsy (ILAE) guidelines (20, 21), with almost half of patients genetically tested after screening for epilepsy surgery (22). Genetic disorders should not preclude surgical indication for epilepsy surgery and can guide the selection of surgical candidates (23). Due to the widely diverse number of different genetic syndromes and mutations that lead to refractory epilepsy, success rates are difficult to estimate, with better outcomes reported in mutations in the mTOR pathway (23).

Our presented patient had an uncommon evolution with drug-resistant epilepsy in Sotos syndrome setting. Despite bilateral mesial temporal lobe been abnormal at investigation (left side showing sclerosis and right side malformation), the detailed investigation depicted a focal onset seizure in left medial temporal lobe due to mesial temporal sclerosis with excellent outcome after resection. Our report is the first one to describe a patient with complete awake seizure control after temporal lobe resection and the second operated patient with Sotos syndrome (9). Although both patients had Sotos syndrome, the patient reported by Bättig et al. (9) was genetically diagnosed with the syndrome after surgical procedure, had a diffuse astrocytoma in the operated temporal lobe and abundant diffuse bilateral sharp waves and polyspikes on interictal EEG, making the cases fundamentally different.

Next steps in health care include optimization of antiseizure medications, sleeping EEG to better categorize the nocturnal seizure event. During follow-up, patient stated no intention of second look surgery based on benefits achieved after first seizure and risks involved to another surgical procedure.

## Conclusion

Epilepsy and, less frequently, drug-resistant seizures are features found in patients suffering from Sotos syndrome. In very selected and investigated cases, surgical resection of epileptogenic brain tissue is safe and feasible, improving patient's quality of life and promoting seizure control.

## Data availability statement

The original contributions presented in the study are included in the article/supplementary material, further inquiries can be directed to the corresponding author.

## Ethics statement

The studies involving human participants were reviewed and approved by IRB of Federal University of São Paulo. The patients/participants provided their written informed consent to participate in this study. Written informed consent was obtained from the individual(s) for the publication of any potentially identifiable images or data included in this article.

## Author contributions

Conception and design: LF, TP, and RS. Acquisition of data: LF and TB. Analysis and interpretation of data: LF, TP, ET, HC, MG, and RS. Drafting the article: LF and TP. Reviewed submitted version of manuscript: TP and RS. Approved the final version of the manuscript on behalf of all authors: LF. All authors contributed to the article and approved the submitted version.
